# Cones structure and seed traits of four species of large‐seeded pines: Adaptation to animal‐mediated dispersal

**DOI:** 10.1002/ece3.6273

**Published:** 2020-04-29

**Authors:** Man‐Yu Zhang, Chang‐Xiang Su, Chang‐Hu Lu

**Affiliations:** ^1^ College of Biology and the Environment Nanjing Forestry University Nanjing China

**Keywords:** adaption, animal‐dispersed pines, coevolution, cone morphology, large‐seeded pines, *Pinus armandi*, *Pinus dabeshanensis*, *Pinus koraiensis*, *Pinus pumila*, seed dispersal, seed morphology

## Abstract

Seed dispersal selection pressures may cause morphological differences in cone structure and seed traits of large‐seeded pine trees. We investigated the cone, seed, and scale traits of four species of animal‐dispersed pine trees to explore the adaptations of morphological structures to different dispersers. The four focal pines analyzed in this study were Chinese white pine (*Pinus armandi*), Korean pine (*P. koraiensis*), Siberian dwarf pine (*P. pumila*), and Dabieshan white pine (*P. dabeshanensis*). There are significant differences in the traits of the cones and seeds of these four animal‐dispersed pines. The scales of Korean pine and Siberian dwarf pine are somewhat opened after cone maturity, the seeds are closely combined with scales, and the seed coat and scales are thick. The cones of Chinese white pine and Dabieshan white pine are open after ripening, the seeds fall easily from the cones, and the seed coat and seed scales are relatively thin. The results showed that the cone structure of Chinese white pine is similar to that of Dabieshan white pine, whereas Korean pine and Siberian dwarf pine are significantly different from the other two pines and vary significantly from each other. This suggests that species with similar seed dispersal strategies exhibit similar morphological adaptions. Accordingly, we predicted three possible seed dispersal paradigms for animal‐dispersed pines: the first, as represented by Chinese white pine and Dabieshan white pine, relies upon small forest rodents for seed dispersal; the second, represented by Korean pine, relies primarily on birds and squirrels to disperse the seeds; and the third, represented by Siberian dwarf pine, relies primarily on birds for seed dispersal. Our study highlights the significance of animal seed dispersal in shaping cone morphology, and our predictions provide a theoretical framework for research investigating the coevolution of large‐seeded pines and their seed dispersers.

## Graphical abstract

We investigated the cone, seed, and scale traits of four species of animal‐dispersed pine trees, and predicted three possible seed dispersal paradigms for animal‐dispersed pines: the first, as represented by Chinese white pine and Dabieshan white pine, relies upon small forest rodents for seed dispersal; the second, represented by Korean pine, relies primarily on birds and squirrels to disperse the seeds; and the third, represented by Siberian dwarf pine, relies primarily on birds for seed dispersal.

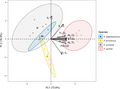

## 1 INTRODUCTION

There are approximately 111 species worldwide in the genus *Pinus* (Fisher, 2001). Pines have two basic seed dispersal modes: wind dispersal, in which the seeds are small with relatively long wings and are carried by the wind away from the canopies of the parent trees (Mwase, Bjørnstad, Ntupanyama, Kwapata, & Bokosi, 2006); and animal dispersal, in which the seeds are relatively large and wingless (or functionally wingless) and unfit for wind dispersal. In the latter case, birds (e.g., nutcrackers and nuthatches) and rodents (e.g., squirrels and chipmunks) act as reciprocal dispersal agents: the animals harvest seeds from the canopy or ground, then scatter‐hoard seeds after satiation to cope with food shortages in the coming winter and spring. Forgotten seed caches are likely to sprout and grow, completing the seed dispersal and increasing the regeneration of large‐seeded pines in temperate forests (Tomback & Linhart, 1990).

Animal‐mediated seed dispersal of *Pinus* species is of great value to the study of coevolution between animals and plants and has therefore been the subject of extensive research (Benkman, 2017; Talluto & Benkman, 2011, 2013); studies have found that animal‐dispersed pines evolved special cone and seed structures or growth patterns to cope with foraging selection pressures by animals. Squirrels are considered to be ineffective seed dispersers, and the cones of pines consumed by squirrels have stronger defense structures where squirrels are in large number, with thicker scales and seed coats, diminished seed numbers, and decreased mass of the nutritious seed kernel (Siepielski & Benkman, 2007). However, in order to promote seed dispersal by nutcrackers, the defense structures of the cones of some pines become relaxed, the cones grow upward, and the opening of the cones is decreased after maturity, increasing the seed retention time and the foraging time for nutcrackers (Tomback & Linhart, 1990). The research of Johnson, Wall, and Borchert (2003) detailed the adaptations of seeds to animal dispersal and wind dispersal by comparing the cone and seed traits of three large‐seeded pines and four wind‐dispersed pines. A recent study at a larger taxonomic scale (all families of Pinales, Taxales, and Podocarpales) found that animal‐dispersed seeds were larger than wind‐dispersed seeds by comparing the seed size and cone morphological characteristics of various coniferous pine species, and revealed that particular cone morphologies are consistently associated with specific ranges in seed size (Leslie, Beaulieu, & Mathews, 2017). The above studies focused on changes in seed dispersal vectors and cone structure within the same species across different distribution areas, or on the morphological differences between animal‐dispersed and wind‐dispersed pines; however, the similarities and differences in cone structure, seed traits, and the adaptive mechanisms behind these traits of different animal‐dispersed pines remain understudied.

China is a center of diversity in *Pinus* plants, with at least 23 species and 10 varieties (Chinese academy of sciences, 2003). Eight of these species of pine (Chinese white pine [*P. armandi*], Korean pine [*P. koraiensis*], Siberian dwarf pine [*P. pumila*], Dabieshan white pine [*P. dabieshanensis*], Hunan white pine [*P. fenzeliana*], Chilgoza pine [*P. gerardiana*], Lacebark pine [*P. bungeana*], and Siberian stone pine [*P. sibirica*]) have seed masses over 90 g (thousand‐grain weight) with short wings or no wings (see Table S1), indicating that they cannot be dispersed effectively by wind (Benkman, 1995b). We predicted that the seeds of these eight species of pine are mainly dispersed by animals. We identified four of these species for which significant research on seed dispersal already exists in the literature (see Table S2), including Dabieshan white pine, Korean pine, Siberian dwarf pine, and Chinese white pine.

The four species of pines utilize different animals for seed dispersal (Table S2), including small forest rodents for Dabieshan white pine, birds and large rodents for Korean pine, birds and various rodents for Chinese white pine, and nutcrackers (*N. caryocatactes*) for Siberian dwarf pine. We predicted that different species of animal‐dispersed pines utilizing similar animals for seed dispersal may show similarities in cone structure and seed traits. We quantified cone, seed, and scale traits and studied the structure of the cones in detail. The parameters representing cone structure were used to compare the similarity of cones and classify the four species of animal‐dispersed pines by principal component analysis and systematic clustering analysis. Finally, we predicted possible evolutionary patterns under which the four species of animal‐dispersed pines emerged.

## 2 METHOD

### 2.1 Pine species and cone collection

We collected cones from Chinese white pine (*P. armandi*), Korean pine (*P. koraiensis*), Siberian dwarf pine (*P. pumila*), and Dabieshan white pine (*P. dabieshanensis*) in the typical distribution areas of these four species (Ma, Zhuang, Chen, & Li, 1992; Peng & Jiang, 1999; Zhang & Cai, 1989; Zhao, 1981).

**FIGURE 1:**
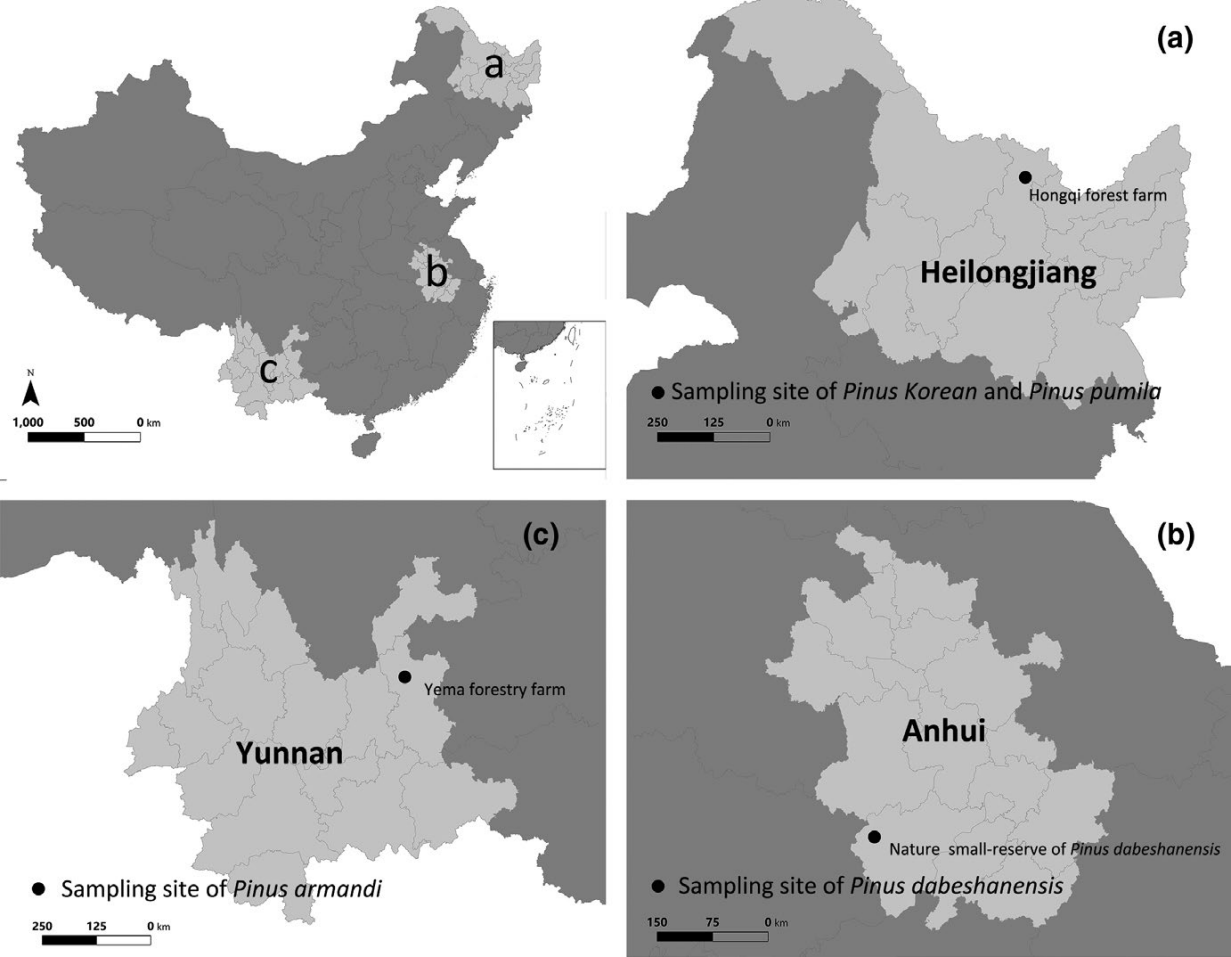
Sampling sites of four focal species of animal‐dispersed pines

Chinese white pine occurs from northern Qinyuan in southern Shanxi Province to central and northwestern Guizhou and Yunnan, and to the lower reaches of the Yarlung Zangbo River in Tibet. We gathered cones at the Chinese Yema forestry farm in Huize County, Yunnan Province (103°38′17″E, 26°10′29″N, 2,700–2,850 m a.s.l.) (Figure 1c). At the Yema forestry farm, Chinese white pine forests are associated with chaparral understory dominated by *Rhododendron decorum*, *Hypericum monogynum*, *Illicium simonsii*, *Pyrus xerophila*, and some ferns and graminaceous plants.

Korean pine is found in the Changbai Mountains and the Xiao Hinggan Mountains. Siberian dwarf pine is distributed throughout the Greater Hinggan Mountains and throughout northeast China. We collected cones of Korean pine and Siberian dwarf pine in the southern region of the Xiao Hinggan Mountains, in the Hegang Forestry Bureau's Hongqi forest farm, and in Heilongjiang Province (129°39′48″E, 48°23′50″N, 577 m a.s.l.) (Figure 1a). This area occurs within the natural range distribution of these trees. Associated flora in the mixed forest included *Quercus mongolica* and *Tilia tuan*.

Dabieshan white pine is primarily distributed in the Dabie Mountains of southwestern Anhui Province and eastern Hubei Province. We collected cones in the Dabieshan white pine nature reserve in Yuexi County, Anhui Province (116°05′30″E, 30°49′24″N, 1,050–1,200 m a.s.l.) (Figure 1b). This site has the largest natural population of Dabieshan white pine in China. Natural populations occur in coniferous mixed forest, and associated vegetation includes *P. taiwanensis*, *Quercus serrata*, *Castanea seguinii*, *Bothrocaryum controversum*, and Rhododendron.

Chinese white pine, Korean pine, and Dabieshan white pine are tall trees; mature plants with cones whose DBH was between 20 and 25 cm were selected as the target sampling plants. Siberian dwarf pine is a clumping species found in shrubby low forest on mountains; we selected mature plants with cones at a height of about 4 m for collection. The cones of the four focal species in the above three sites were intensively sampled (Figure 1). A total of 50 fresh cones were collected from 10 trees (*n* = 5 per tree) between October and November in 2017 and were used for measurements of each species of these four animal‐dispersed pines (Benkman, 1995a).

After picking, the cones were naturally dried and preserved. We excluded very large and small cones to avoid confounding the results of intraspecific differences and optimized 5 cones for each species of pine from which to measure the parameters. Three to five cones per tree are needed accurately assess the variation among trees in most cone and seed traits (Garcia, Siepielski, & Benkman, 2009). We extracted the seeds from the cones and removed stunted seeds by submergence method (Zhang, Chen, & Qi, 2015). After air drying, 300 seeds of each species of pine were randomly selected to prepare for the measurement of the seed parameters. While extracting the seeds, we peeled the scales from the top, middle, and bottom portions of the cones (5 scales from each cone, 20 scales from each species) to prepare for the measurement of the scale parameters.

### 2.2 Cone and seed traits measurement

The cone traits (cone length, cone diameter, number of seeds, and peduncle diameter) (Benkman & Siepielski, 2004; Tikhonova, Tarakanov, Tikhonova, Barchenkov, & Ekart, 2014) of 5 cones were recorded to 0.1 mm using an electronic caliper. The seed traits (seed length, seed diameter, seed height, and seed coat thickness) of 20 seeds were also recorded 0.1 mm with an electronic caliper (Aslam, Reshi, & Siddiqi, 2010; Tikhonova et al., 2014). Seed weight was taken in three replicates and expressed as the hundred‐grain weight (*n* = 100), recorded to 0.1 mg using an analytical balance. We estimated the volume of the seeds by measuring the seed length (*l*), width (*w*), and height (*h*) of 20 seeds as ellipsoids:

$$V=\frac{3}{4}\times \left(\pi \times \frac{l}{2}\times \frac{w}{2}\times \frac{h}{2}\right)$$

We used a three‐dimensional variance method (Wang, Huang, Pu, Zhou, & Lu, 2014) to measure the seed shape: variance (*S* ^2^) was calculated according to the seed length (*l*), width (*w*), and height (*h*) of 20 seeds, using the following formula:

$$S^{2}=\frac{3\left(l^{2}+w^{2}+h^{2}\right)-{\left(l+w+h\right)}^{2}}{3^{2}}$$

Smaller variance indicates that the shape of seed is closer to spherical; otherwise, the seed is flatter in the shape.

### 2.3 Scale traits measurement

**FIGURE 2:**
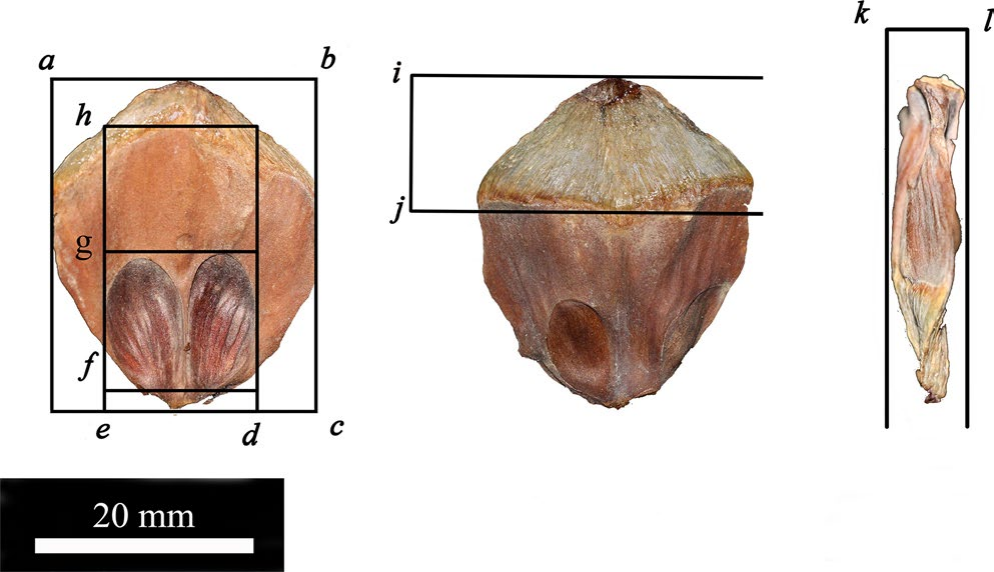
Parameters of morphology of scales (ab‐AW, bc‐TL, kl‐ST, ij‐AL, ed‐FW, eg‐IL, fg‐FL)

Five mature cones of each species of pine were selected, and 5 scales were stripped from the top, middle, and bottom of each cone, for a total of 100 samples. Seven morphological parameters of the scales were measured with a Vernier caliper at a precision of 0.1 mm (Figure 2), including apophysis width (AW), total length (TL), fossa width (FW), ingrowth length (IL), fossa length (FL), apophysis length (AL), and scale thickness (ST) (Tikhonova et al., 2014). The parameters AW, TL, ST, and AL represent the size of scale, and the parameters FW, IL, and FL indicate the size of the seed growth site on the scale.

### 2.4 Statistical analysis

To eliminate the influence of the size of the cone on the morphological parameters of the different species of pine, 7 parameters of the scales were divided by the corresponding cone length (CL) or cone diameter (CD) for standardization; in addition, the following parameters were added: apophysis width/scale thickness (AL/ST), ingrowth length/scale thickness (IL/ST), and fossa length/ingrowth length (FL/IL), for a total of 10 cone structure parameters.

Two methods (principal component analysis and systematic clustering analysis) were used to analyze the 10 cone structure parameters. We used principal component analysis to extract two principal components, rotated their factors to obtain independent variables with different high load coefficients (high correlation), extracted the scores of the first and second principal components, and drew the scatter diagram (Figure 4). The four species of animal‐dispersed pines could be classified using plots of the scores of their first and second principal components. Systematic clustering analysis was used to classify the four species of animal‐dispersed pines, using the same 10 parameters (Figure 5).

All analyses were conducted in R (3.5.1) using the stats package for data testing and one‐way ANOVA, vegan for data conversion, stats for principal component analysis and cluster analysis, and the packages factoextra, ggdendro, and ggplot2 for cluster tree and principal component analysis diagrams.

## 3 RESULTS

### 3.1 Cones and seed traits

**FIGURE 3:**
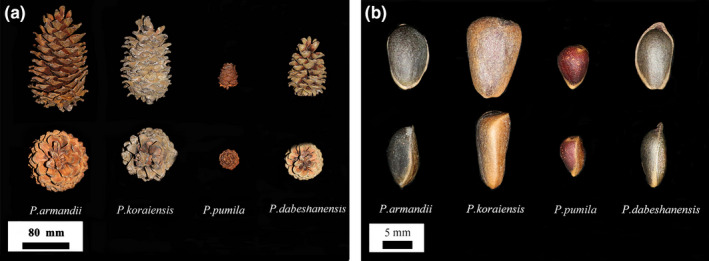
Comparison of cones and seeds of four species of animal‐dispersed pines

There were significant differences among the cone traits of the four species of animal‐dispersed pines (Table 1). The cones of Chinese white pine were the largest, followed by Korean pine, Dabieshan white pine, and Siberian dwarf pine. Korean pine produced an average of 143.00 ± 11.47 seeds per cone, Chinese white pine produced 140.60 ± 13.41 seeds per cone, Dabieshan white pine produced 123.80 ± 3.56 seeds per cone, and Siberian dwarf pine produced 49.20 ± 3.70 seeds per cone. The peduncle diameter of Chinese white pine was the thickest, followed by that of Korean pine, Dabieshan white pine, and Siberian dwarf pine. From the top view of the cones, the apexes of the scales of Chinese white pine and Dabieshan white pine displayed obvious outward retroflexion, while those of Korean pine and Siberian dwarf pine were relatively flat and the basal scales were tightly wrapped (Figure 3a).

**TABLE 1:** Cones and seed traits of four species of animal‐dispersed pines

Parameters	*n*	*Pinus armandii*	*Pinus koraiensis*	*Pinus pumila*	*Pinus dabeshanensis*	*p*
Cone length (mm)	5	159.16 ± 9.09a	140.17 ± 1.50b	47.17 ± 0.97d	108.29 ± 2.12c	<.01
Cone diameter (mm)	5	94.90 ± 13.79a	81.23 ± 9.52b	35.83 ± 4.70d	69.50 ± 4.28c	<.01
Number of seeds	5	140.60 ± 13.41a	143.00 ± 11.47a	49.20 ± 3.70c	123.80 ± 3.56b	<.01
Peduncle diameter (mm)	5	11.37 ± 0.70a	9.33 ± 0.16b	4.18 ± 0.23d	7.02 ± 0.57c	<.01
Seed length (mm)	20	13.54 ± 0.69b	17.11 ± 0.96a	9.18 ± 0.69d	12.33 ± 0.77c	<.01
Seed width (mm)	20	9.02 ± 0.69b	11.26 ± 0.88a	6.44 ± 0.53d	7.00 ± 0.53c	<.01
Seed height (mm)	20	7.06 ± 0.57b	7.75 ± 0.62a	5.44 ± 0.57c	5.29 ± 0.35c	<.01
*V* (mm^3^)	20	453.70 ± 16.15b	785.12 ± 27.17a	168.8 ± 6.24d	240.12 ± 8.21c	<.01
*S* ^2^	20	0.04 ± 0.01c	0.05 ± 0.01b	0.03 ± 0.01d	0.06 ± 0.01a	<.01
Hundred‐grain weight (g)	100	31.54 ± 1.3b	58.51 ± 0.8a	12.18 ± 0.27d	19.00 ± 0.31c	<.01
Seed coat thickness (mm)	20	0.54 ± 0.01b	0.92 ± 0.05a	0.41 ± 0.04c	0.36 ± 0.03d	<.01

Note

Mean ± *SE*, *p* < .001 was a significant difference. Mean values followed by the different letter (a, b, c, and d) are significantly different as determined by multiple range test, and there is no significant difference between groups with the same letter.

The seed traits of the four pine species of pine were significantly different (Table 1), with the exception of the seed height of Siberian dwarf pine and Dabieshan white pine. Korean pine produced seeds with largest value of “V,” followed by the seeds of Chinese white pine and Dabieshan white pine, while the seeds of Siberian dwarf pine were the smallest. The seeds of Dabieshan white pine were the flattest, while the seeds of Korean pine, Chinese white pine, and Siberian dwarf pine were more and more spherical. Korean pine had the heaviest hundred‐grain weight, followed by Chinese white pine, Dabieshan white pine, and Siberian dwarf pine. The seed coat of the Korean pine was 0.92 ± 0.05 mm, which was the thickest, followed Chinese white pine. Although the seeds of Siberian dwarf pine were the smallest, their seeds' coat was not the thinnest; the pine with the thinnest seed coat was Dabieshan white pine. Tiny woody wings were observed at the top of the seeds of Dabieshan white pine, while the seeds of other three pines were wingless and displayed slight ridges on both sides and on the top (Figure 3b).

### 3.2 Scales traits

We measured the parameters AW, TL, ST, and AL of the scales and compared the differences among the four species of pine (Table 2). Chinese white pine had the largest scales, and the apophysis was also large; the scale thickness was moderate. The scale size of Korean pine was second only to that of Chinese white pine, but it displayed the longest apophysis and the thickest scales. The scales of Siberian dwarf pine were the smallest, but the scales were thicker than those of Dabieshan white pine. The scales of Dabieshan white pine were smaller than those of Chinese white pine and Korean pine and larger than those of Siberian dwarf pine and were the thinnest of all measured scales.

**TABLE 2:** Scales traits of four species of animal‐dispersed pines

Parameters	*n*	*Pinus armandii*	*Pinus koraiensis*	*Pinus pumila*	*Pinus dabeshanensis*	*p*
AW (mm)	25	29.70 ± 1.43a	28.48 ± 2.23b	17.41 ± 1.10d	20.87 ± 0.23c	<.01
TL (mm)	25	40.30 ± 4.24a	36.99 ± 2.13b	15.20 ± 1.15d	27.35 ± 1.08c	<.01
ST (mm)	25	8.26 ± 0.93b	11.15 ± 0.99a	8.35 ± 0.49b	5.60 ± 0.61c	<.01
AL (mm)	25	17.15 ± 2.36a	18.03 ± 1.59a	9.64 ± 1.39c	12.78 ± 1.04b	<.01
FW (mm)	25	16.19 ± 0.92b	20.18 ± 0.73a	12.44 ± 0.66c	12.19 ± 0.66c	<.01
IL (mm)	25	34.45 ± 4.44a	25.86 ± 1.91b	12.75 ± 1.02d	23.02 ± 1.27c	<.01
FL (mm)	25	15.18 ± 0.79b	17.52 ± 1.19a	9.05 ± 0.64d	13.77 ± 1.04c	<.01

Note

Mean ± *SE*, *p* < .001 was a significant difference. Mean values followed by the different letter (a, b, c, and d) are significantly different as determined by multiple range test, and there is no significant difference between groups with the same letter.

We measured the parameters FW, IL, and FL of the scales and compared the differences among the four pine species (Table 2). The fossa of Chinese white pine was large and long, but the proportion (FL/IL) bearing seed was small. The fossa of Korean pine was the largest among the four species. The fossa of Siberian dwarf pine was shorter than that of the other three species, but was wider than that of Dabieshan white pine. The fossa of Dabieshan white pine was the narrowest but was longer than that of Siberian dwarf pine.

### 3.3 Classification of four species of animal‐dispersed pines

**FIGURE 4:**
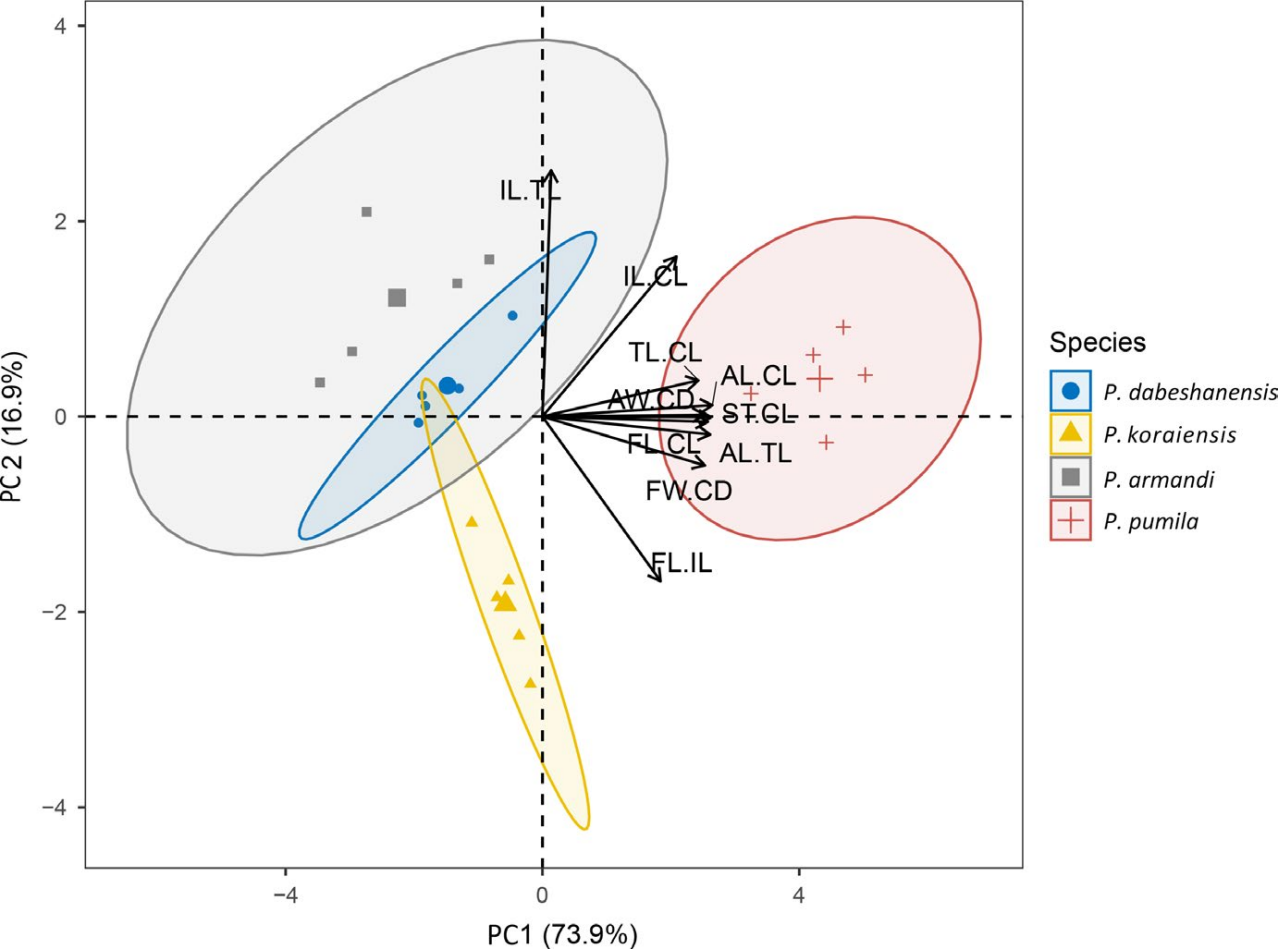
The scatter plot of morphological parameters of cones in four species of animal‐dispersed pines

According to the first principal component (Figure 4), Siberian dwarf pine can be easily distinguished from the other three species of pine; according to the second principal component, Korean pine can be easily distinguished from the other three species of pine. In addition, the first and the second principal components overlapped between Chinese white pine and Dabieshan white pine, indicating a high degree of similarity in the structure of the cones between the two species.

**FIGURE 5:**
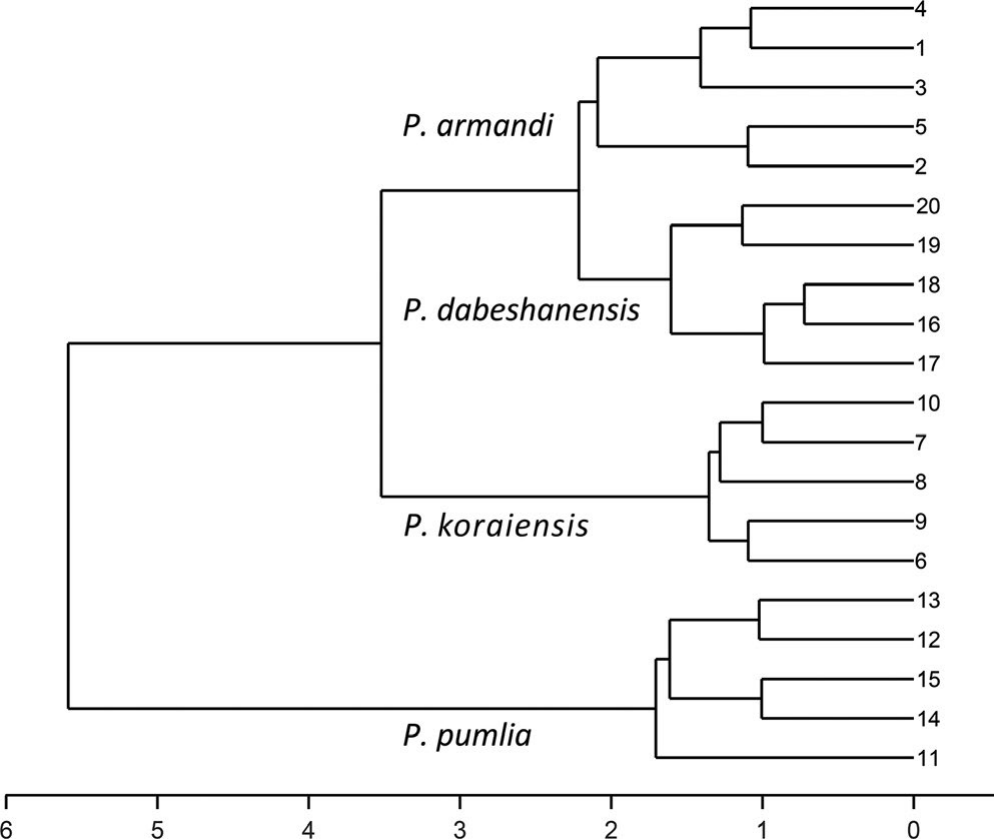
Cluster tree of four species of animal‐dispersed pines. *Y* axis, 1–20, represents the sample cone, and the parameters of each species of pine come from five cones, 20 cones in total

The cluster tree (Figure 5) illustrates that the four species of animal‐dispersed pines can be well distinguished. When the height is 3, the four species of animal‐dispersed pines can be divided into three categories, and Chinese white pine and Dabieshan white pine could be classified into one category. When the height is 4, the four species can be divided into two categories, and Siberian dwarf pine is observably different from the other three species.

## 4 DISCUSSION

This study found that there are significant differences in cone and seed traits between the species. Chinese white pine had the largest cones, followed by Korean pine, Dabieshan white pine, and Siberian dwarf pine; Chinese white pine also had the thickest peduncle, followed by Korean pine, Siberian dwarf pine, and Dabieshan white pine. The seeds of Korean pine were far larger than the seeds of the other three pines and showed the same order as the size of cones. The scale of Korean pine was the thickest, followed by Siberian dwarf pine, Chinese white pine, and Dabieshan white pine. For Chinese white pine and Dabieshan white pine, the primary dispersers include small forest rodents (see Table S2) and the structures of their cones are very similar. Korean pine and Siberian dwarf pine seeds are dispersed by various birds, and large rodents and nutcrackers, respectively (see Table S2); their cone structures differ greatly from the other two pines and vary significantly from each other.

### 4.1 Effects of cone traits on seed dispersal by animals

The cone traits like size and peduncle diameter often determine that animals use the cones for forage, and the fate of cones at maturity. Tong (2011) found that the larger size of Korean pine cones deterred squirrels' predation. Chinese white pine and Korean pine cones are relatively large among the four species studied here, inhibiting rodent predation prior to cone ripening. A thicker core of vascular tissue in the peduncle decreased the rate of cone of Limber pine (*P. flexilis*) removal by red squirrels (Benkman, Balda, & Smith, 1984). Chinese white pine and Korean pine have thicker peduncles than Dabieshan white pine and Siberian dwarf pine, preventing squirrels from biting off the cones and consuming them.

### 4.2 Effects of seed traits on seed dispersal by animals

Seed traits influence the foraging behavior and seed dispersal efficiency of animals. Some studies have shown that large seeds increase the probability of both predation and successful dispersal (Alcantara & Rey, 2003; Wang & Ives, 2017), although most studies were performed on individuals and populations of a single species. Large seeds provide more seed kernel, providing a higher profitability for animals. In our research, the seeds of Korean pine were the largest, heaviest, and the richest in nutrients, which may attract animals to forage upon them. The seeds of Chinese white pine and Dabieshan white pine had similar sizes, and the seeds of Siberian dwarf pine were the smallest, which may be related to their growth in barren environments (Zhao, 1981). Seed coat thickness is another important factor in limiting predation; for example, Coulter pine (*P. coulteri*) evolved seeds with a very thick seed coat, dissuading nutcrackers and resulting in cones that could only be eaten by rodents (Borchert, Johnson, Schreiner, & Wall, 2003). Of the four focal species of pine in our study, the seeds of Chinese white pine, Korean pine, and Siberian dwarf pine had relatively thick seed coats, perhaps limiting their predation by dispersers and promoting dispersal efficiency. The seed coat of Dabieshan white pine was significantly thinner than that of the other three pines, making the seeds extremely vulnerable to predation and destruction; this finding is consistent with the high rate of predation found in the only other study on seed dispersal of Dabieshan white pine (Su, Zhong, & Lu, 2018). Dabieshan white pine is a rare species endemic to China (Wang et al., 2014), and its regeneration is very low in the wild (Xu, 2016; Xu, et al., 2012), which may be related to its thin seed coat.

### 4.3 Effects of scale traits on seed dispersal by animals

Scale traits determine the fate of seeds after cone ripening. The time of the closure or opening of the scales may influence the foraging time and the dispersal efficiency (Dawson, Vincent, & Rocca, 1997; Soons & Bullock, 2008). If the scales open slowly when the cones ripen, seeds can easily fall out of the cones and be eaten readily by predators (Mcmaster & Zedler, 1981). The cones of Chinese white pine and Dabieshan white pine open after maturation, coupled with their downward growth pattern this causes the seeds to fall to the ground quickly. The cones of Korean pine and Siberian dwarf pine exhibit delayed opening after maturation, and the seeds can be kept in the cones for extended periods of time. For mature cones, large and thick scales may increase seed feeding difficulty and reduce dispersal efficiency (Borchert et al., 2003; Johnson et al., 2003). Large and thick scales indicate high allocation of energy to defensive structures, and corresponding small seeds; however, thick scales impede predation (Benkman, 1999; Mezquida & Benkman, 2005; Parchman & Benkman, 2008). In our research, the value of ST represents the thickness of scales, and the ST of Korean pine and Siberian dwarf pine was both large, indicating that scale thickness may reduce the foraging efficiency of animals.

### 4.4 Seed dispersal patterns of four animal‐dispersed pines

The results of the principal component analysis and cluster analysis showed that Chinese white pine and Dabieshan white pine were very similar, while the structures of Korean pine, Siberian dwarf pine, and Chinese white pine were very different (Figures 4 and 5). According to the analysis of 10 comprehensive parameters of the cones, scales, and seeds, we predicted three possible seed dispersal modes for the four animal‐dispersed pines.

The first seed dispersal pattern, represented by Chinese white pine and Dabieshan white pine, relies primarily upon small forest rodents for seed dispersal. The scales open after maturity, the seeds fall readily to the ground, and the seed coat of these two pines is relatively thin, making the cone structure and seed traits of these two pines suitable for dispersal by small forest rodents. Studies on seed dispersal in Chinese white pine showed that a variety of animals feed on the seeds, including the Eurasian nutcracker (*Nucifraga caryocatactes*), Chevrier's field mouse (*Apodemus chevrieri*), Sichuan field mouse (*A. latronum*), and various small forest rodents (see Table S2). In our study, we speculated that the Eurasian nutcracker may not be the primary seed disperser and that small forest rodents of many species may play an important role in dispersing seeds. The cones of Dabieshan white pine grow downward from the branches (Chinese academy of sciences, 2003) and do not provide a stable foothold, impeding the foraging of birds; additionally, the scales reduce the foraging time (Tomback & Linhart, 1990). The only available study about seed dispersal in Dabieshan white pines (Su et al., 2018) reported that only two forest rodents were found to feed on the seed, and we therefore speculate that birds are not the main seed dispersers of Dabieshan white pine, while the small forest rodents that feed on the ground might act as the main seed dispersers.

The second pattern, which is represented by Korean pine, primarily relies upon birds and squirrels to disperse seeds. Korean pine has the largest seeds among the four studied species and attracts many animals to forage upon its seeds, including scatter‐hoarding dispersers such as the Eurasian nutcracker, ordinary nuthatch, squirrels, and chipmunks (Lu, 2001, 2002). The scales open slightly after the ripening of the cones, and birds and large rodents must break or bite the scales off to obtain the seeds. The process of seed dispersal in Korean pines has been studied in detail, and these studies have indicated that the Eurasian nutcracker is a very effective disperser; squirrels are the secondary dispersers, but when the number of squirrels increases, Korean pines enhance their energy input to defensive structures (e.g., thick scale and seed coat) and produce smaller and fewer seeds (Hayashida, 1998; Miyaki, 1987). This adaptation to reduce foraging efficiency and prevent predation upon the seeds by inefficient seed dispersers is similar to that of the Limber pine (*P. flexilis*) in North America and the Aleppo pine (*P. halepensis*) in the Mediterranean region (Benkman, 1995a; Mezquida & Benkman, 2005).

The third pattern, which is represented by Siberian dwarf pine, primarily relies on Eurasian nutcrackers for seed dispersal. The cone structure and seed traits of Siberian dwarf pine show remarkable divergence from the other three species of pine. The size of the cone and seed is the smallest, the shape of seeds is globular, and the seeds and scales are closely combined, traits that allow birds to force open the scales and obtain the seeds. The only two studies (Kajimoto, 2002; Kajimoto, Onodera, Ikeda, Daimaru, & Seki, 1998) on seed dispersal in Siberian dwarf pines showed that nutcrackers are their seed dispersers, although the study site was in Japan. Additional field research in China is needed to clarify the details of the dispersal of Siberian dwarf pines.

## 5 CONCLUSION

Our study shows that there are significant differences in the size of cones, seeds, and scales of the four species of animal‐dispersed pines, but some similarities emerged in the cone structure of two species. This suggests that species with similar seed dispersal strategies exhibit similar morphological adaptions. We propose three possible evolutionary patterns of animal‐dispersed pines: the first, represented by Chinese white pine and Dabieshan white pine, relies on small forest rodents for seed dispersal; the second, represented by Korean pine, mainly relies on birds and squirrels to disperse seeds; and the third, represented by Siberian dwarf pine, mainly relies on birds for seed dispersal. Thus, our study highlights the value of animal seed dispersal in shaping cone morphology, and our predictions provide new clues and trajectories for the study of the coevolution of large‐seeded pines and their seed dispersers.

The results of our research are, however, based on small‐scale spatial sampling of each species and incorporated existing seed dispersal reports. Our findings ignore the possible interspecific differences of species within a wide area and the variation in seed disperser assemblages. Little research has been conducted on individual pine species and their dispersal strategies, and we therefore recommend more long‐term and large‐scale field research on the seed dispersal strategies of large‐seeded pines to reveal more accurate evolutionary trajectories.

## CONFLICT OF INTEREST

None declared.

## AUTHOR CONTRIBUTIONS

**Man‐Yu Zhang:** Conceptualization (equal); investigation (equal); methodology (equal); software (equal); visualization (lead); writing‐original draft (lead); writing–review and editing (lead). **Chang‐Xiang Su:** Conceptualization (equal); investigation (lead). **Chang‐Hu Lu:** Conceptualization (equal); project administration (lead).

## Supporting information

Table S1‐S2

## Data Availability

Morphological data on traits of cones, seeds, and scales and cone structure is available from figshare dataset: https://doi.org/10.6084/m9.figshare.11959284.v1

## References

[ece36273-bib-0001] Alcantara, J. M. , & Rey, P. J. (2003). Conflicting selection pressures on seed size: Evolutionary ecology of fruit size in a bird‐dispersed tree, *Olea europaea* . Journal of Evolutionary Biology, 16(6), 1168–1176. 10.1046/j.1420-9101.2003.00618.x 14640408

[ece36273-bib-0002] Aslam, M. , Reshi, Z. A. , & Siddiqi, T. O. (2010). Variability in cone and seed characteristics among plus trees of blue pine (Pinus Wallichiana A. B. Jackson) in the Kashmir Himalaya, India. International journal of pharma and bio sciences, 1(4), 197–206. 10.3906/tar-0902-26

[ece36273-bib-0003] Benkman, C. W. (1995a). The impact of tree squirrels (Tamiasciurus) on Limber pine seed dispersal adaptations. Evolution, 49(4), 585–592. 10.1111/j.1558-5646.1995.tb02295.x 28565136

[ece36273-bib-0004] Benkman, C. W. (1995b). Wind dispersal capacity of pine seeds and the evolution of different seed dispersal modes in pines. Oikos, 73(2), 221–224. 10.2307/3545911

[ece36273-bib-0005] Benkman, C. W. (1999). The selection mosaic and diversifying coevolution between crossbills and Lodgepole pine. The American Naturalist, 153(Suppl), S75–S91. 10.1086/303213 29578779

[ece36273-bib-0006] Benkman, C. W. (2017). Matching habitat choice in nomadic crossbills appears most pronounced when food is most limiting. Evolution, 71(3), 778–785. 10.1111/evo.13146 27925171

[ece36273-bib-0007] Benkman, C. W. , Balda, R. P. , & Smith, C. C. (1984). Adaptations for seed dispersal and the compromises due to seed predation in Limber pine. Ecology, 65(2), 632–642. 10.2307/1941426

[ece36273-bib-0008] Benkman, C. W. , & Siepielski, A. M. (2004). A keystone selective agent? Pine squirrels and the frequency of serotiny in Lodgepole pine. Ecology, 85(8), 2082–2087. 10.1890/04-0177

[ece36273-bib-0009] Borchert, M. , Johnson, M. , Schreiner, D. , & Wall, S. B. V. (2003). Early postfire seed dispersal, seedling establishment and seedling mortality of *Pinus coulteri* (D. Don) in central coastal California, USA. Plant Ecology, 168(2), 207–220. 10.1023/A:1024447811238

[ece36273-bib-0010] Chinese academy of sciences (2003). Flora of China (Vol. 7). Beijing, China: Science Press.

[ece36273-bib-0011] Dawson, C. , Vincent, J. F. V. , & Rocca, A.‐M. (1997). How pine cones open. Nature, 390(6661), 668–668. 10.1038/37745

[ece36273-bib-0012] Fisher, R. F. (2001). Ecology and biogeography of pinus. Forest Ecology and Management, 152(1), 339–340. 10.1043/0363-6445-27.3.636

[ece36273-bib-0013] Garcia, R. , Siepielski, A. M. , & Benkman, C. W. (2009). Cone and seed trait variation in whitebark pine (Pinus albicaulis; Pinaceae) and the potential for phenotypic selection. American Journal of Botany, 96(5), 1050–1054. 10.3732/ajb.0800298 21628255

[ece36273-bib-0014] Hayashida, M. (1998). Seed dispersal by red squirrels and subsequent establishment of Korean pine. Forest Ecology and Management, 28(2), 115–129. 10.1016/0378-1127(89)90064-9

[ece36273-bib-0015] Johnson, M. , Wall, S. B. , & Borchert, M. (2003). A comparative analysis of seed and cone characteristics and seed‐dispersal strategies of three pines in the subsection Sabinianae. Plant Ecology, 168(1), 69–84. 10.1023/A:1024470224134

[ece36273-bib-0016] Kajimoto, T. (2002). Factors affecting seedling recruitment and survivorship of the Japanese subalpine stone pine, *Pinus pumila*, after seed dispersal by nutcrackers. Ecological Research, 17(4), 481–491. 10.1046/j.1440-1703.2002.00505.x

[ece36273-bib-0017] Kajimoto, T. , Onodera, H. , Ikeda, S. , Daimaru, H. , & Seki, T. (1998). Seedling establishment of subalpine stone pine (*Pinus pumila*) by Nutcracker (Nucifraga) seed dispersal on Mt. Yumori, Northern Japan. Arctic and Alpine Research, 30(4), 408–417. 10.2307/1552014

[ece36273-bib-0018] Leslie, A. B. , Beaulieu, J. M. , & Mathews, S. (2017). Variation in seed size is structured by dispersal syndrome and cone morphology in conifers and other nonflowering seed plants. New Phytologist, 216(2), 429–437. 10.1111/nph.14456 28185279

[ece36273-bib-0019] Lu, C. H. (2001). Effect of rodents on seed dispersal. Chinese Journal of Ecology, 20(6), 56–58.

[ece36273-bib-0020] Lu, C. H. (2002). Hoarding behavior of eurasian nutcracker (*Nucifraga caryocatact*) and its role in seed dispersal of Korean pine (*Pinus koraiensis*). Acta Zoologica Sinica, 48(03), 317–321. 10.3969/j.issn.1674-5507.2002.03.004

[ece36273-bib-0021] Ma, J. L. , Zhuang, L. W. , Chen, D. , & Li, J. D. (1992). The geographical distribution of Korean pines. Journal of Northeast Forestry University, 20(5), 40–48.

[ece36273-bib-0022] Mcmaster, G. S. , & Zedler, P. H. (1981). Delayed seed dispersal in *Pinus torreyana* (Torrey Pine). Oecologia, 51(1), 62–66. 10.1007/BF00344654 28310311

[ece36273-bib-0023] Mezquida, E. T. , & Benkman, C. W. (2005). The geographic selection mosaic for squirrels, crossbills and Aleppo pine. Journal of Evolutionary Biology, 18(2), 348–357. 10.1111/j.1420-9101.2004.00846.x 15715841

[ece36273-bib-0024] Miyaki, M. (1987). Seed dispersal of the Korean pine, *Pinus koraiensis*, by the red squirrel. Sciurus Vulgaris. Ecological Research, 2(2), 147–157. 10.1007/BF02346923

[ece36273-bib-0025] Mwase, W. F. , Bjørnstad, Å. , Ntupanyama, Y. M. , Kwapata, M. B. , & Bokosi, J. M. (2006). Phenotypic variation in fruit, seed and seedling traits of nine *Uapaca kirkiana* provenances found in Malawi. Southern African Forestry Journal, 208(1), 15–21. 10.2989/10295920609505257

[ece36273-bib-0026] Parchman, T. L. , & Benkman, C. W. (2008). The geographic selection mosaic for ponderosa pine and crossbills: A tale of two squirrels. Evolution, 62(2), 348–360. 10.1111/j.1558-5646.2007.00295.x 17999725

[ece36273-bib-0027] Peng, Z. H. , & Jiang, Z. H. (1999). Pinus dabeshanensis and its origin. Beijing, China: China Forestry Publishing House.

[ece36273-bib-0028] Siepielski, A. M. , & Benkman, C. W. (2007). Convergent patterns in the selection mosaic for two north American bird‐dispersed pines. Ecological Monographs, 77(2), 203–220. 10.1890/06-0929

[ece36273-bib-0029] Soons, M. B. , & Bullock, J. M. (2008). Non‐random seed abscission, long‐distance wind dispersal and plant migration rates. Journal of Ecology, 96(4), 581–590. 10.1111/j.1365-2745.2008.01370.x

[ece36273-bib-0030] Su, C. X. , Zhong, Z. F. , & Lu, C. H. (2018). Role of animals in the natural population regeneration of Pinus dabeshanensis. Acta Ecologica Sinica, 38(17), 6194–6203. 10.5846/stxb201710281933

[ece36273-bib-0031] Talluto, M. V. , & Benkman, C. W. (2011). The Role of the American Red Squirrel (*Tamiasciurus hudsonicus*) in the Evolution of Serotiny in Lodgepole Pine (*Pinus contorta*). University of Wyoming National Park Service Research Center Annual Report, 33(1), 155–160.

[ece36273-bib-0032] Talluto, M. V. , & Benkman, C. W. (2013). Landscape‐scale eco‐evolutionary dynamics: Selection by seed predators and fire determine a major reproductive strategy. Ecology, 94(6), 1307–1316. 10.2307/23436151 23923494

[ece36273-bib-0033] Tikhonova, I. V. , Tarakanov, V. V. , Tikhonova, N. A. , Barchenkov, A. P. , & Ekart, A. K. (2014). Population variability of cones and seeds of scots pine by phenes of color and traits‐indices in the south of Siberia. Contemporary Problems of Ecology, 7(1), 60–66. 10.1134/S1995425514010156

[ece36273-bib-0034] Tomback, D. F. , & Linhart, Y. B. (1990). The evolution of bird‐dispersed pines*. Evolutionary Ecology, 4(3), 185–219. 10.1007/BF02214330

[ece36273-bib-0035] Tong, G. (2011). Feeding on Korean pine seeds and effects of seed hoarding on Korean pines in Liangshui Nature Reserve. Chinese Journal of Wildlife, 32, 75–79. 10.19711/j.cnki.issn2310-1490.2011.02.005

[ece36273-bib-0036] Wang, L. H. , Huang, Q. F. , Pu, F. G. , Zhou, M. S. , & Lu, F. C. (2014). Interspecific relationship between Pinus dabeshanensis and dominant species in the community at Tianma National Nature Reserve. Resources and Environment in the Yangtze Basin, 23(07), 960–964. 10.1870/cjlyzyyhj201407004

[ece36273-bib-0037] Wang, B. , & Ives, A. R. (2017). Tree‐to‐tree variation in seed size and its consequences for seed dispersal versus predation by rodents. Oecologia, 183(3), 751–762. 10.1007/s00442-016-3793-0 28000021

[ece36273-bib-0038] Xu, G. M. (2016). Existing problems in and strategies for the conservation of *Pinus dabeshanensis* population in Dawanggou ravine of Yuexi county. Anhui Forestry Science and Technology, 42(1), 69–72. 10.3969/j.issn.2095-0152.2016.01.020

[ece36273-bib-0039] Xu, Y. Y. , Wang, L. , Song, X. G. , Zhang, X. W. , & Guo, C. Y. (2012). Research progress in *Pinus dabeshanensis* . Journal of Huaibei Normal University(Natural Science), 33(3), 55–58. 10.3969/j.issn.2095-0691.2012.03.013

[ece36273-bib-0040] Zhang, A. X. , Chen, Y. , & Qi, L. Q. (2015). Study of quality testing method for seeds of *Silybum marianum* . Chinese Traditional and Herbal Drugs, 46, 580–583. 10.7501/j.issn.0253-2670.2015.04.023

[ece36273-bib-0041] Zhang, Y. Y. , & Cai, H. Q. (1989). Geographical distribution and community ecology of *Pinus armandii* . Hunan Forestry Science & Technology, 4, 5–8.

[ece36273-bib-0042] Zhao, G. Y. (1981). Investigation about Distribution of *Pinus sibirica* in Daxing'an mountains and northwest limit of *Pinus koraiensis* in China. Journal of Northeast Forestry University, 2, 31–40. 10.13759/j.cnki.dlxb.1981.02.004

