# Supplementary material for: Cones structure and seed traits of four species of large‐seeded pines: Adaptation to animal‐mediated dispersal
Source: Ecol Evol. 2020 Apr 29;10(12):5293–301. doi: 10.1002/ece3.6273 (PMC7319130; doi:10.1002/ece3.6273)
Supplement: Supplementary file 1 — Table S1‐S2 [file ECE3-10-5293-s001.pdf]

Supplement

TABLE 1 The seed traits of pines distributed in China.

Pinus		Species	Seed mass (TGW/g)	Seed size(mm)		Wings(mm)	Reference
				length	width		
Subgen Strobus	1	*P. fenzeliana*	172-215	12-15	6-7	2-4	State Forestry Bureau (2001); Chinese academy of sciences (2003)
	2	*P. gerardiana*	412	17-23	5-7	0	State Forestry Bureau (2001); Chinese academy of sciences (2003)
	3	*P. dabeshanensis*	130-150	11-13	6-7	1-2	Chinese academy of sciences (2003); Xiang et al. (2016)
	4	*P. koraiensis*	350-600	12-17	8-12	0	State Forestry Bureau (2001); Chinese academy of sciences (2003)
	5	*P. pumila*	121-130	7-10	4-7	0	State Forestry Bureau (2001); Chinese academy of sciences (2003)
	6	*P. bungeana*	140-165	10-12	5-6	4-6	State Forestry Bureau (2001); Chinese academy of sciences (2003)
	7	*P. armandii*	230-290	10-15	6-10	0	State Forestry Bureau (2001); Chinese academy of sciences (2003)
	8	*P. sibirica*	220-245	7-12	5-6	0	State Forestry Bureau (2001); Chinese academy of sciences (2003)
	9	*P. wallichiana*	40-50	7-8	4-5	20-30	State Forestry Bureau (2001); Chinese academy of sciences (2003)
	10	*P. kwangtungensis*	78.6-79	8-12	-	8-12	Duan & Chen (1992)
	11	*P. morrisonicola*	-	8-10	5-6	15-20	State Forestry Bureau (2001); Chinese academy of sciences (2003)
	12	*P. wangii*	55-70	7-12	5-6	16	State Forestry Bureau (2001); Chinese academy of sciences (2003)
	13	*P. squamata*	19-20	-	-	16	Lu et al. (1999)
Subgen Pinus	14	*P. densata*	11-26	4-6	3-4	9-18	State Forestry Bureau (2001); Chinese academy of sciences (2003)
	15	*P. densiflora*	7-12	4-7	-	10-12	State Forestry Bureau (2001); Chinese academy of sciences (2003)
	16	*P. kesiya*	13-21	5-6.5	3-4	12-15	State Forestry Bureau (2001); Chinese academy of sciences (2003)
	17	*P. henryi*	18-20	6-8	4	12-14	State Forestry Bureau (2001); Chinese academy of sciences (2003)
	18	*P. latteri*	24-35	5-8	4-5	7-14	Han et al. (2014)
	19	*P. massoniana*	8.5-13.6	4-6	2.5-3	15-20	State Forestry Bureau (2001); Chinese academy of sciences (2003)
	20	*P. tabuliformi*	32-50	6.5-9.5	3.5-5.0	10-20	State Forestry Bureau (2001); Chinese academy of sciences (2003)
	21	*P. yunnanensis*	11-18	3.5-6.0	-	12-15	State Forestry Bureau (2001); Chinese academy of sciences (2003)

22	*P. taiwanensis*	10-16	4-6	2.0-3.5	7-12	State Forestry Bureau (2001); Chinese academy of sciences (2003)
23	*P. roxburghii*	110-150	8-12	5-7	25	Ghildiyal et al. (2009)

REFERENCE

Chinese academy of sciences. (2003). *Flora of China* (Vol. 7). Beijing: Science Press.

Duan, X. P., & Chen, W. J. (1992). A preliminary study on seed dormancy physiology of Pinus kwangtungensis. *Hunan Forestry Science & Technology*(03), 25-30.

Ghildiyal, S. K., Sharma, C. M., & Gairola, S. (2009). Environmental variation in seed and seedling characteristics of Pinus roxburghii Sarg. From Uttarakhand, India. *Applied Ecology and Environmental Research, 7*(2), 121-129. https://doi.org/10.15666/aeer/0702_121129

Han, C., Xu, J. M., & Tong, Q. (2014). Study on the selection of provenance / family of Pinus australis introduction at Seedling Stage. *Chinese Agricultural Science Bulletin, 30*(19), 7-12.

Lu, S. J., Wang, J. W., & Zhu, H. P. (1999). A Study on Some Bilogical Characteristics of Pinus squamata. *Journal of Southwest Forestry University(Natural Sciences), 19*(02), 86-89.

State Forestry Bureau. (2001). *Seeds of Woody Plants in China*: Beijing:China Forestry Publishing House.

Xiang, X. Y., Wu, G. L., & Wang, Z. G. (2016). Comparison of seed characteristics of Pinus dabeshaneses in different seed stands. *Journal of Anqing Normal University(Natural Science Edition), 22*(3), 110-112.

TABLE 2 Seed dispersers of eight potential animal-dispersed pines in China.

Pinus spp.	Dispersers	References
P. fenzeliana	No report	None
P. gerardiana	No report	None
P. dabeshanensis	Rodentia: *Sciurotamias davidianus*, *Apodemus sylvaticus*	Su et al. (2018)
P. koraiensis	Rodentia: *Tamias sibiricus*, *Sciurus vulgaris*<br>Passeriformes: *Sitta europaea*, *Nucifraga caryocatactes*	Miyaki (1987); Hutchins et al. (1996); Lu (2002, 2006); Yi et al. (2008); Yang & Yi (2011)
P. pumila	Passeriformes: *Nucifraga caryocatactes*	Kajimoto et al. (1998); Kajimoto (2002)
P. bungeana	No report	None
P. armandii	Rodentia: *Sciurotamias davidianus, Garrulus glandarius*, *Sciurus vulgaris*, *Tamias sibiricus,Dremomys pernyi, Callosciurus erythraeus, Tamiops swinhoei, Apodemus latronum, A. chevrieri, A. draco, A. agrarius, A. sylvaticus, A. peninsulae, Niviventer fulvescens,N. niviventer, Rattus nitidus, Microtus fortis, Eothenomys custos*<br>Passeriformes: *Nucifraga caryocatactes*	Chen & Chen (2011); Wang et al. (2012); Yu et al. (2014)
P. sibirica	No report	None

Reference

Chen, F., & Chen, J. (2011). Effects of Pinus armandii seed size on rodents caching behavior and it's spatio-temporal variations. *Zoological Research, 32*(4), 435-441. https://doi.org/10.3724/SP.J.1141.2011.04435

Hutchins, H. E., Hutchins, S. A., & Liu, B. (1996). The role of birds and mammals in Korean pine (Pinus koraiensis) regeneration dynamics. *Oecologia, 107*(1), 120-130. https://doi.org/10.1007/BF00582242

Kajimoto, T. (2002). Factors affecting seedling recruitment and survivorship of the Japanese subalpine stone pine, Pinus pumila, after seed dispersal by nutcrackers. *Ecological Research, 17*(4), 481-491. https://doi.org/10.1046/j.1440-1703.2002.00505.x

Kajimoto, T., Onodera, H., Ikeda, S., Daimaru, H., & Seki, T. (1998). Seedling establishment of subalpine stone pine (Pinus pumila) by Nutcracker (Nucifraga) seed dispersal on Mt. Yumori, Northern Japan. *Arctic and alpine research, 30*(4), 408-417.

Yu, F., Wang, D. X., Yi, X. F., Shi, X. X., Huang, Y. K., Zhang, H. W., & Zhang, X. P. (2014). Does animal-mediated seed dispersal facilitate the formation of Pinus armandii-Quercus aliena var. acuteserrata forests? *Plos One, 9*(2), e89886. https://doi.org/10.1371/journal.pone.0089886

Lu, C. H. (2002). Hoarding behavior of eurasian nutcracker (Nucifraga caryocatact) and its role in seed dispersal of Korean pine (Pinus koraiensis). *Acta Zoologica Sinica, 48*(03), 317-321. https://doi.org/10.3969/j.issn.1674-5507.2002.03.004

Lu, C. H. (2006). Roles of animals in seed dispersal of Pinus: A review. *Chinese Journal of Ecology, 25*(5), 557-562.

Miyaki, M. (1987). Seed dispersal of the Korean pine, Pinus koraiensis, by the red squirrel, Sciurus vulgaris. *Ecological Research, 2*(2), 147-157. https://doi.org/10.1007/BF02346923

Su, C. X., Zhong, Z. F., & Lu, C. H. (2018). Role of animals in the natural population regeneration of Pinus dabeshanensis. *Acta Ecologica Sinica, 38*(17), 6194-6203. https://doi.org/10.5846/stxb201710281933

Wang, B., Wang, G., & Chen, J. (2012). Scatter-hoarding rodents use different foraging strategies for seeds from different plant species. *Plant Ecology, 213*(8), 1329-1336. https://doi.org/10.1007/s11258-012-0091-8

Yang, Y. Q., & Yi, X. F. (2011). Effectiveness of Korean Pine (Pinus Koraiensis) Seed Dispersal by Small Rodents in Fragmented and Primary Forests. *Polish Journal of Ecology, 59*(2), 413-422. https://doi.org/10.1007/s10211-011-0101-1

Yi, X. F., Xiao, Z. S., & Zhang, Z. B. (2008). Seed dispersal of Korean pine Pinus koraiensis labeled by two different tags in a northern temperate forest, northeast China. *Ecological Research, 23*(2), 379-384. https://doi.org/10.1007/s11284-007-0392-x
